# Effect of corporate social responsibility performance on dividend pay-out: Role of corporate governance quality

**DOI:** 10.3389/fpsyg.2022.883952

**Published:** 2022-08-08

**Authors:** Zahid Maqbool, Ammar Abid, Aamir Inam Bhutta

**Affiliations:** ^1^Department of Business Administration, Riphah International University, Faisalabad, Pakistan; ^2^Department of Management Sciences, COMSATS University Islamabad, Lahore, Pakistan; ^3^Lyallpur Business School, Government College University, Faisalabad, Pakistan

**Keywords:** corporate social responsibility, corporate governance, dividend policy, mutual funds, Pakistan

## Abstract

The goal of this study was to analyze the influence of corporate social responsibility on dividend pay-out while considering the role of corporate governance quality on mutual funds operating in Pakistan. This study used a two-step system generalized method of moments (GMM) to control not only endogeneity problems caused by inclusion of firm-specific variables, but also the endogeneity caused by dividend pay-out selection. The findings are that mutual funds that engage in higher levels of corporate social responsibility pay greater dividends. The quality of corporate governance not only has a strong positive impact on mutual fund’s dividend pay-outs, but also moderates the association between dividend pay-out and corporate social responsibility. Furthermore, differences exist between socially responsible Islamic and conventional mutual funds in terms of dividend pay-out policy. These findings imply that the quality of corporate governance performs a substantial role in dividend decisions. Policymakers and regulators should also encourage asset management firms to improve corporate governance quality and engage in more socially responsible activities, which can lead to improved fund performance and dividend pay-out.

## Introduction

In the latest decades, corporate social responsibility (CSR) has seen significant changes. Firms are now approaching CSR as a tactic to build a soft image and to enhance their competitive advantage, which could be helpful to boost the firm’s value (Robinson et al., 2011). The studies of the nexus between CSR and financial performance have spawned CSR research in the finance arena (Wang et al., 2016; Awaysheh et al., 2020; Barauskaite and Streimikiene, 2021). Previous studies link CSR performance with various corporate decisions such as financial leverage (Harjoto, 2017; Sheikh, 2019), idiosyncratic risk (Mishra and Modi, 2013), cash holding (Arouri and Pijourlet, 2017), and pay-out of dividends (Cheung et al., 2018; Benlemlih, 2019; Sheikh et al., 2021), all of which help companies increase value by reducing information asymmetry (Dhaliwal et al., 2011).

Over the last decade, mutual funds have experienced enormous growth, capturing the attention of both individual and institutional investors all over the world. Today, mutual funds are considered an important investment alternative for people who do not have enough knowledge, competence, or experience to create their own investment portfolio of financial assets and properly invest their money (Afza and Rauf, 2009; Clare et al., 2019). Investors are concerned not only about mutual fund performance, but also about the frequency with which dividends are paid (Harris et al., 2015; Naveed, 2021). A recent astonishing development and rise in socially responsible mutual funds makes the topic “whether CSR affects the pay-out policies of mutual funds” relevant, in addition to regulatory distinctions in mutual funds’ dividend payments compared with other corporations. According to the US Social Investing Forum (USSIF), sustainable investment accounts for 33% of the $12 trillion industry in 2018.

The number of socially responsible funds is growing continuously following the global agenda of sustainable development. Fund managers, on the contrary, are dramatically increasing their investments in socially responsible enterprises with better financial prospects and CSR performance (Bauer et al., 2005; El Ghoul and Karoui, 2017; Durán-Santomil et al., 2019). Investors in socially responsible firms (firms with higher CSR engagements) receive higher dividends, according to empirical research, than investors in less socially responsible firms (Samet and Jarboui, 2017; Cheung et al., 2018; Benlemlih, 2019; Sheikh et al., 2021).

According to the authors’ knowledge, this is the earliest efforts to enrich the literature through linking CSR and dividend policy in the mutual fund business. Furthermore, the moderating influence of corporate governance (CG) on nexus between CSR and dividend policy is being studied, because prior research has shown that enterprises’ dividend policy is sensitive to the quality of CG (Jiraporn et al., 2011; Abor and Fiador, 2013; Elmagrhi et al., 2017). Previously, somewhat related to our study Cheung et al. (2018) and Benlemlih (2019) documented a positive impact of CSR performance on dividend pay-out of US firms. Although these studies have improved our understanding of the consequences of CSR performance, the focus is on the developed economies and dividend policy at the firm level, thus ignoring the emerging markets. This study departs from the existing studies by examining the impact of CSR performance on dividend pay-out of mutual funds in an emerging economy. The dynamics of emerging markets and mutual funds are somewhat in stark contrast to the firms in the developed markets. The emerging markets are characterized by weak regulatory enforcement, inefficient legal system, poor investor protection, information opacity, and continued policy uncertainty (Abid et al., 2018; Saeed and Zamir, 2021). Furthermore, investors of mutual funds are more concerned about dividend payments compared with firm-level investors (Naveed, 2021). These market differences and investors’ perceptions in mutual funds may have different outcomes of CSR performance on dividend policy of mutual funds and thus merit further investigation in a different context. In this paper, we focus on the context of Pakistan, an emerging economy that adopts a stakeholders’ centric corporate governance model, and have a highly concentrated family business ownership structure marked by the weak protection of minority investors and weak-form information environment.

One of the two main conflicting explanations could elucidate the link between CSR activities and mutual funds’ dividend pay-out. On the one hand, managers who invest in higher socially responsible companies may be investing in stocks with strong financial fundamentals, which can lead to better fund performance (El Ghoul and Karoui, 2017). Mutual fund’s dividend pay-outs are projected to rise as fund performance improves. CSR initiatives can increase profits for several reasons, including enhanced stakeholder relationships, more effective management, lower transaction costs, and acquiring a competitive edge. Because of their increased earning capability and superior return performance linked with socially responsible investing, mutual funds may pay higher dividends. Investing primarily in companies that practice CSR, on the other hand, may limit the number of investment alternatives available and raise monitoring costs (Cortez et al., 2009). In this situation, CSR would have a negative influence on fund returns and performance, resulting in decreased mutual fund’s dividend pay-outs.

We evaluate the impact of CSR performance on dividend distribution using a sample of 185 equity funds and 1,592 observations between 2009 and 2019. First, this study shows that stronger CSR performance is linked to higher dividend pay-outs. Our findings show that the fund’s CSR score is positively connected to dividend pay-out but adversely associated with the dividend frequency. Furthermore, we discover that fund’s CG quality moderates the association between portfolio CSR score and dividend distribution and frequency.

This research adds to the current knowledge in several aspects. First, there is a line of inquiry into the effect of CSR on firm performance, company value, company policies, and financial decisions. This paper participates in the CSR discussion by providing evidence on the impact of CSR engagement on dividend distribution policy, one of the most divisive topics in corporate finance. Second, this is the first study that we are aware of to look at the influence of CSR performance on a mutual fund’s dividend pay-out. This paper also shows that in a developing market setting, there is a link between CG quality and mutual fund’s dividend pay-out, which means that CG should be reinforced to stimulate investment channels. Finally, this paper looks at the role of mutual funds’ CG quality in moderating the relationship between CSR and dividend pay-out, adding to the body of knowledge on mutual funds’ CG quality. One notable contribution in the context of mutual funds is that we also examine the asymmetric impact of CSR performance on dividend policy of conventional and Islamic mutual funds.

The remaining sections of the paper are organized as follows. The literature and the construction of hypotheses are discussed in Section “Related literature and hypotheses development.” The study strategy and data and sample, the model, and variable measurement are all found in Section “Methodology and data.” Section “Results and discussion” delves into the findings and analyses, while Section “Conclusion” wraps up the paper.

## Related literature and hypotheses development

It is well-established fact that CSR investments have a major impact on market valuation (Hong and Kacperczyk, 2009; Yoo and Lee, 2018). According to the market valuation model, companies with stronger CSR are likely to increase their future cash flows and predicted profitability which can then be allocated to shareholders as dividend payments. CSR efforts can also help a company gain a competitive advantage, develop stakeholder relationships, manage operations more efficiently, and lower transaction costs, among other things. All these initiatives are aimed at increasing revenue and profit margins. Increased profitability and related cash flows logically result in a higher dividend pay-out.

There are two opposing arguments that could be used to explain the association between CSR and mutual fund’s dividend pay-out. First, due to the limited number of options accessible in this category, investing solely in SRI funds could be costly and would result in excessive monitoring expenses, which would have hurt mutual fund performance and result in lower dividend distributions. The counter viewpoint is that funds that apply SRI screening criteria select companies with a lengthy track record of financial strength and profitability as well as solid long-term prospects. As a result, it can be anticipated that funds that invest in companies with aggressive CSR participation will earn a higher return while posing a reduced risk. Because mutual fund investors care about dividend payments as well as profits, increased dividend pay-outs can result from stronger fund performance and decreased risk associated with CSR. They also reveal that corporations with a high level of corporate social responsibility pay out more consistent dividends.

The higher dividend payments in higher socially responsible funds are consistent with the signaling theory of dividends and communicate strong signals relating to the funds’ efficient investments as well as help to create a positive image among the fund’s investors. As the legitimacy prospects require justifications and explanations by stakeholders from firms with respect to ethical approaches in CSR investments, Moratis (2016) provides physiological explanations of credible CSR messages delivered by the organizations to the suspicious and distrustful internal and external stakeholders. Costly signaling theory from evolutionary psychology (BliegeBird and Smith, 2005) is more appropriate to justify our case. It suggests that the honesty and impact of signals depend on efforts and financial cost associated with the generation of signals. Firms are ready to carry such burdens in lieu of that signal-receiving parties recognize such costs more credible and feel more comfortable to investment in such firms as compared to the firms not able to carry similar responsibilities. Applying this to CSR–dividend nexus, funds investing in more socially responsible firms may pay more frequent and higher dividends to signal their investors that their investment in CSR anticipates stronger future financial performance and has solid bases (Lys et al., 2015).

Until now, the majority of research on the relationship between CSR and dividend policy has focused on non-mutual fund’s dividend pay-outs, neglecting mutual fund dividend disbursements. Samet and Jarboui (2017) and Badru and Qasem (2021), for example, show a positive association between CSR and dividend payment using the total pay-out (both dividend payment and share repurchase). Benlemlih (2019) recently observed that in the United States, companies with a high CSR pay more dividends than companies with a low CSR. Cheung et al. (2018) and Ni and Zhang (2019) discovered that CSR had a positive impact on the dividend pay-out ratio but no significant impact on the propensity to pay dividends for US-listed businesses. More recently, Salah and Amar (2022) also provide evidence of positive impact of CSR practices on dividend policy in the French setting and found that individual dimensions of CSR have a positive relationship with a dividend pay-out. Contrarily, Saeed and Zamir (2021) found a negative relationship between CSR and dividend pay-out in emerging markets and owed the negative association with the higher institutional ownership and funding of growth opportunities.

Overall, the research suggests that corporate social responsibility has a positive impact on dividend distribution. However, given mutual fund investors’ interest in dividend payments, these studies do not look at mutual fund’s dividend pay-outs.

We hypothesize the following based on the reasoning discussed and empirical evidence of the association between CSR and dividend payment:

**H1:** *CSR is positively associated with dividend payout of mutual funds.*

Managers tend to act for their own personal gain at the expense of shareholders due to the separation of control and ownership in the firm. The agency problem is exacerbated by a lack of good corporate governance. Grossman and Hart (1980) studied the dividend distribution patterns of businesses and found that paying dividends reduces the free cash flows available to managers, lessening the agency conflict between shareholders and management. Similarly, firms with large free cash flows, according to Jensen (1986) and Chang et al. (2016), have negative net present value investment projects. Dividend payments to shareholders on a regular basis lower free cash flow and help to alleviate free cash flow concerns. Dividends alleviate the agency problem by lowering the amount of free cash flow that can be used for personal advantage by opportunistic managers. Corporate governance serves a moderating role in resolving the agency conflict by establishing checks and balances between management and shareholders.

For a variety of reasons, there is a high probability to pay fewer dividends from companies with poor CG practices. The evidence shows that companies operating in civil law countries paid lower dividends than companies operating in common law countries, according to La Porta et al. (2000). They attribute this conduct to a lack of investor protection and a weak corporate governance framework in civil law countries. As a result, the dividend pay-out may be linked to the level of corporate governance.

According to different reasons, companies with inadequate corporate governance are more likely to pay lesser dividends. In this regard, La Porta et al. (2000) discovered that firms in civil law countries paid lower dividends than those in common law countries. In civil law countries, they ascribe this practice to a lack of investor protection and a weak corporate governance system. As a result, the quality of corporate governance may be linked to the dividends pay-out. Therefore, it is believed that the better the disclosure of the CG, the bigger the dividend pay-out. This argument is strengthened by the fact that shareholders are protected and can easily receive dividend payments from free cash flows in a good CG system. Managers with a poor CG structure are more likely to use free cash flow for expropriation purpose at the expense of dividends payment. As a result, a strong corporate governance structure leads to increased dividend pay-outs.

The prior papers on dividend distribution policy (La Porta et al., 2000; Jiraporn et al., 2011; Yarram and Dollery, 2015; Elmagrhi et al., 2017) suggest that strong CG firms pay regular dividends and have fewer agency problems. These studies suggest that corporations with better CG pay higher dividends to shareholders, implying a relationship between CG and dividend policy.

Some academics have discovered a correlation CSR and dividend pay-outs (Barnea and Rubin, 2010; De Cesari and Ozkan, 2015). Similarly, recent research by Yarram and Dollery (2015) and Elmagrhi et al. (2017) demonstrated a link between CG quality and dividend distribution strategies in recent studies. According to the research above, organizations with better CG quality and CSR transparency offer higher dividends to shareholders to attract more capital. Therefore, the following two hypotheses are being suggested:

**H2a:** *CG quality has a positive impact on a mutual fund’s dividend pay-out.*

**H2b:** *The association between CSR and mutual fund’s dividend pay-out is positively moderated by CG quality*.

## Methodology and data

### Study variables

Previous studies used a variety of proxies to assess the mutual funds’ pay-out policy. The first proxy is the dividend payment ratio (total dividend to total income), which was used by Jabbouri (2016); Elmagrhi et al. (2017), and Naveed (2021) to calculate the dividend pay-out. The second measure is the dividend frequency, which indicates how many times a fund pays a dividend each year (Yarram and Dollery, 2015). Our study uses both proxies.

#### Corporate social responsibility index

Following Borgers et al. (2015) and El Ghoul and Karoui (2017, 2019), this study uses the matching fund holding approach considering the each stock’s characteristics to assess the mutual fund’s CSR. In the first step, we identify the firms on which mutual fund portfolio is based and calculate the weight of investment in each stock. In the second step, we construct the CSR disclosure index including seven dimensions such as health sector, natural disaster, environmental issues, employee’s welfare, donation for the educational sector, product/services statements, and other donations. First, we compute the total CSR count based on the above seven dimensions. The score is calculated by using the binary numbers (0/1). If the firm discloses the item of the above-discussed CSR dimensions in the annual report, then code 1; otherwise, 0. After assigning a binary number to each CSR item, the total score for the company is calculated by adding the scores of all items. This is commonly used method to construct the CSR index in the literature (Reverte, 2009; Benlemlih, 2014; Majeed et al., 2015; Ehsan et al., 2018). Finally, the following fund-year level equation is used to calculate a CSR score of each mutual fund:


(1) $$CSR_{j}{,}_{t}=\sum_{i=1}^{N_{j}{,}_{t}}\omega_{i}{,}_{j}{,}_{t}\times CSR_{i}{,}_{t}$$

where i is the weight of company in which fund j has investment during year t, N is the total number of companies in which fund j has investment during year t, and CSR is the score of company in which fund j has investment during year t.

#### Corporate governance quality

This study creates a CG index comprising several governance rules in accordance with the SECP^1^ ’s CG recommendations to examine the role of CG quality in the dividend distribution decisions of socially responsible enterprises. Previous studies, such as Samaha et al. (2012); Javaid and Saboor (2015), and Naveed et al. (2020a), developed a CG index that included a variety of governance mechanisms to assess the CG quality. Following the above studies, a comprehensive CG index is constructed where the higher total score represents the highest quality. The quality of CG is measured by the total score of this index. The fund obtains a score of 1 if it follows the CG provisions; otherwise, it receives a score of 0. The total CG score of a specific fund each year is calculated by adding the individual scores of all governance parameters. Because the governance index consists of 32 provisions, the maximum CG score of any fund in any given year is 32. A higher score indicates better corporate governance, and vice versa.

#### Control variables

The fund and managerial characteristics (managerial experience, manager experience, and management fee) are used as control variables. Table 1 shows the measurement and the data sources of the control variables.

**TABLE 1:** Detail of control variables’ definitions.

Variables	Definitions	Source
F_Size	F_Size represents the fund size defined as natural log of all net assets.	Ferreira et al., 2013
F_Age	F_Age signifies fund age calculated as a total number of years since a fund is established.	Makni et al., 2015
Exp_Ratio	Exp_ratio is the total expenses divided total net assets.	Makni et al., 2015
MGT_Fee	MGT_Fee denotes the ratio of fee charged by management	Bauer et al., 2006
M_Edu	M_Edu symbolizes the managers’ education which is a dummy variable assigned as value 1 if manager has professional qualification (FCA, ACCA, etc.); otherwise, 0.	Naidenova et al., 2015
M_Exper	M_Exper is the managers’ professional experience in years in asset management industry.	Naidenova et al., 2015
Perf	Perf signifies the Jenson alpha-based performance of fund, which is calculated as follows: *R*_*it*_ – *R*_*ft*_ = α_*i*_ + β_1_(*R*_*mt*_ – *R*_*ft*_) + ε_*it*_	Reddy et al., 2017

### Data and sample

There are 284 mutual funds in Pakistan, with 137 conventional funds and 147 Islamic funds. After excluding funds with missing data and recently launched funds with fewer than 12-month net asset values, our final sample contains 185 mutual funds, with 100 conventional funds and 85 Islamic funds. This sample is free of survivorship bias, and it spans 11 years, from 2009 to 2019. This study used data from 2009 to 2019 because the Islamic mutual funds have seen tremendous growth during the last decade. Furthermore, the Islamic mutual funds in Pakistan were limited before this period and the data were not available. The study could not include the data of 2020 in the sample because the measurement of corporate social responsibility (CSR) performance variable requires the manual content analysis of CSR disclosures, which is quite laborious and time-consuming. At the time of content analysis of CSR disclosures, the annual reports of the year 2020 were not publicly available. The data on fund features such as management fee and expense ratio are collected through monthly fund managers’ reports. The rest of the control variables are derived from data from the funds’ annual reports. Annual reports of all firms in which mutual funds have investments are downloaded to create the CSR index. The funds’ annual reports were obtained from the mutual funds’ websites.

### Model

Dynamic panel methods are common techniques to address the issues related to panel data. Among the dynamic panel methods, a two-step system generalized method of moments (GMM) is the most robust technique which controls not only the endogeneity problems simultaneously generated by the inclusion of firm-specific variables but also endogeneity caused by the selection of dividend pay-out. The system GMM overcomes the problem of weak instruments of difference GMM estimation with the use of suitable first difference and lag value as instruments. The fitness of GMM estimations depends on conditions in which the number of instruments should be less than the number of groups or endogenous. Therefore, we use the xtabond2 command in Stata to calculate two-step system GMM regressions.

The following dynamic equations have been developed to test the hypotheses of the study using panel data:


$$D\,i\,v\,i\,d\,e\,n\,d_{i\,t}=\alpha_{0}+\beta_{1}\,L.D\,i\,v\,i\,d\,e\,n\,d_{i\,t}+\beta_{2}\,C\,S\,R_{i\,t}+\beta_{3}\,P\,e\,r\,f_{i\,t}+$$

$$\beta_{4}\,F\,\_\,S\,i\,z\,e_{i\,t}+\beta_{5}\,E\,x\,p\,\_\,R\,a\,t\,i\,o_{i\,t}+\beta_{6}\,M\,G\,T\,\_\,F\,e\,e_{i\,t}+$$

$$\beta_{7}\,M\,\_\,E\,d\,u\,c\,a\,t\,i\,o\,n_{i\,t}+\beta_{8}\,M\,\_\,E\,x\,p\,e\,r\,i\,e\,n\,c\,e_{i\,t}+$$

(2) $$\beta_{9}\,F\,\_\,A\,g\,e_{i\,t}+\varepsilon_{i\,t}$$

$$D\,i\,v\,i\,d_{i\,t}=\alpha_{0}+\beta_{1}\,L.D\,i\,v\,i\,d\,e\,n\,d_{i\,t}+\beta_{2}\,C\,S\,R_{i\,t}+\beta_{3}\,C\,G\,\_\,I\,n\,d\,e\,x_{i\,t}$$

$$+\beta_{4}\,C\,S\,R_{i\,t}*C\,G\,\_\,I\,n\,d\,e\,x_{i\,t}+\beta_{5}\,P\,e\,r\,f_{i\,t}+\beta_{6}\,F\,\_\,S\,i\,z\,e_{i\,t}$$

$$+\beta_{7}\,E\,x\,p\,\_\,R\,a\,t\,i\,o_{i\,t}+\beta_{8}\,M\,G\,T\,\_\,F\,e\,e_{i\,t}+$$

$$\beta_{9}\,M\,\_\,E\,d\,u\,c\,a\,t\,i\,o\,n_{i\,t}+\beta_{10}\,M\,\_\,E\,x\,p\,e\,r\,i\,e\,n\,c\,e_{i\,t}+$$

(3) $$\,\beta_{11}\,F\,\_\,A\,g\,e_{i\,t}+\varepsilon_{i\,t}$$

where *Dividend*_*it*_ indicates the fund dividend policy at the time t as measured by the dividend pay-out ratio and dividend frequency, *CSR*_*it*_ refers to the fund’s CSR score, *CG_Index_*it*_* indicates the overall score of CG quality index, and *CSR*_*it*_ × *CG*_*Index*_*it*_ is the interaction term between CSR and CG quality. The definitions of control variables are mentioned in Table 1.

Table 2 describes the descriptive statistics of the outcome variables, namely, dividend pay-out ratio and frequency to pay dividend, along with predictor CSR and other control variables. The mean identifies the central tendency of data, while the standard deviation shows the variation in the mean. The mean values of the dependent variable’s dividend pay-out ratio and frequency to pay are 0.33 and 1.746, respectively, with standard deviations of 0.326 and 2.204, which indicate the Pakistani mutual fund’s dividend pay-out ratio is 33 percent and the funds on average pay dividend more than once in a year. The average CSR score for the fund is 0.346 percent, with a standard deviation of 0.101 percent, while the mean CG quality score is 26.211 percent, with a standard deviation of 2.354 percent. The typical values for fund size, age, performance, and expense ratio are 13.94, 7.564, -0.144, and 2.674, respectively. Management fees, manager experience, and education had mean values of 1.525, 10.43, and 0.359, respectively, for managerial attributes.

**TABLE 2:** Descriptive statistics of the variables.

Variable	Obs	Mean	Std. dev.	Min	Max
Pay-out	1,491	0.332	0.33	0	0.985
Dividend_Frequency	1,491	1.746	2.204	0	12
CSR	1,491	0.347	0.115	0.082	0.624
CG_Index	1,491	26.211	2.354	21	32
Perf	1,491	–0.144	0.752	–2.261	1.931
F_Size	1,491	13.941	1.349	11.53	17
Exp_Ratio	1,490	2.674	1.579	0.18	6.32
MGT_Fee	1,491	1.525	0.61	0	3
M_Edu	1,491	0.359	0.48	0	1
M_Exper	1,491	10.431	4.779	2	21
F_Age	1,491	7.564	5.269	1	30

The correlation analysis result of all variables is shown in Table 3. Control variables do not have a high correlation among themselves, according to the overall study. The fund size and expense ratio have a maximum correlation of 0.53. As a result, we can conclude that multicollinearity between variables is not a big issue. The variables’ variance inflation factor (VIF) value is also less than 3.

**TABLE 3:** Correlation matrix.

Variables	(1)	(2)	(3)	(4)	(5)	(6)	(7)	(8)	(9)	(10)	(11)
(1) Pay-out	1.00										
(2) Dividend_Frequency	0.53*	1.00									
(3) CSR	0.02	–0.05	1.00								
(4) CG_Index	0.39*	0.35*	–0.02	1.00							
(5) Perf	0.34*	0.29*	0.03	0.51*	1.00						
(6) F_Size	–0.05*	0.04	–0.07*	0.01	0.13*	1.00					
(7) Exp_Ratio	–0.12*	–0.22*	–0.04	–0.14*	–0.28*	–0.23*	1.00				
(8) MGT_Fee	0.07*	–0.00	–0.01	–0.10*	–0.24*	–0.22*	0.52*	1.00			
(9) M_Edu	0.08*	0.02	0.07*	0.10*	0.15*	0.09*	–0.07*	0.02	1.00		
(10) M_Exper	0.36*	0.44*	0.00	0.34*	0.26*	–0.01	–0.18*	0.00	–0.03	1.00	
(11) F_Age	0.00	–0.04	–0.04	0.02	–0.03	0.03	0.29*	0.23*	–0.07*	–0.05	1.00

*p < 0.05.

## Results and discussion

Table 4 summarizes the results of two-step system GMM regressions used to test hypotheses 1 and 2. Model 1 and model 3 report the regressions to test hypothesis 1 using two proxies of dividend, namely, pay-out ratio and frequency to pay dividend. Model 2 and model 4 test the moderating effects of CG on association between dividend and CSR. The significant coefficients of lagged dependent variables confirm the dynamic nature of the models. Moreover, AR1, AR2, and Hensen J. statistics confirm the validity of two-step system GMM regressions in all models.

**TABLE 4:** Corporate social responsibility (CSR) and dividend pay-out: role of corporate governance.

	(M-1)	(M-2)	(M-3)	(M-4)
Variables	Pay-out	Pay-out	Dividend_Frequency	Dividend _Frequency
L. Pay-out	0.132***	0.120***		
	(11.516)	(9.564)		
L. Dividend_Frequency			0.010	0.023***
			(1.359)	(2.971)
CSR	0.251***	–2.030***	–2.337***	12.796***
	(3.710)	(–2.878)	(–6.394)	(2.663)
CG_Index		0.028***		0.425***
		(2.650)		(6.873)
CSR × GC_Index		0.088***		–0.580***
		(3.238)		(–3.256)
Perf	0.107***	0.049***	0.907***	0.782***
	(16.857)	(5.548)	(10.261)	(9.143)
F_Size	–0.028***	–0.015***	–0.001	0.006
	(–7.580)	(–4.050)	(–0.059)	(0.256)
Exp_Ratio	–0.046***	–0.043***	–0.244***	–0.225***
	(–11.702)	(–10.489)	(–10.459)	(–8.191)
MGT_Fee	0.117***	0.166***	0.308***	0.407***
	(7.214)	(10.399)	(5.421)	(7.309)
M_Edu	0.040***	0.014	–0.090	–0.088
	(4.087)	(1.429)	(–1.250)	(–1.148)
M_Exper	0.019***	0.013***	0.147***	0.122***
	(18.443)	(11.699)	(17.734)	(13.800)
F_Age	0.013***	0.010***	0.034***	0.026***
	(8.534)	(7.021)	(3.596)	(2.651)
Year effects	Yes	Yes	Yes	Yes
Constant	0.163**	–0.661**	0.443	–10.690***
	(2.392)	(–2.304)	(1.155)	(–6.393)
Observations	1,305	1,305	1,305	1,305
Number of sr	185	185	185	185
AR1 (Pr > z)	–7.957 (0.00)	–7.950 (0.00)	–7.069 (0.00)	–7.385 (0.00)
AR2 (Pr > z)	–0.292 (0.77)	–0.707 (0.48)	–0.429 (0.67)	0.771 (0.44)
Hansen J. (Prob > Chi)	126.0 (0.19)	119.4 (0.275)	109.8 (0.33)	108.8 (0.30)

z-statistics in parentheses ***p < 0.01, **p < 0.05, *p < 0.1.

The findings are consistent with the market valuation approach, which shows that CSR participation is likely to boost the predicted cash flows and profitability (Sasongko et al., 2020), which can be distributed as dividend payments. CSR efforts can aid in gaining a competitive advantage, enhancing stakeholder relationships, improving management, and lowering transaction costs. Our results imply that mutual funds using SRI screening criteria are selecting firms for investment with a sustainable financial and profitability performance. Therefore, mutual funds are more likely to have a superior return performance and lower risk, which can translate into higher dividend payments. The control variables such as F_Age, M_Exper, M_Educ, and MGT_Fee have a positive and significant coefficient, which indicate that funds having a higher performance paid a higher dividend to shareholders. On the contrary, large funds and funds with a higher expense ratio pay significantly lower dividend.

Model 2 of Table 4 reports the findings of equation 3 with dividend payment as the dependent variable. The CG index coefficients are 0.028 significant at *p* < 0.01. In economic terms, a one-standard-deviation increase in corporate governance quality raises dividend payments by 19.85%. This finding backs with hypothesis 2a, which states that funds with superior corporate governance pay larger dividends to shareholders. This is in line with Naveed et al. (2020b) prior research, which found that funds with superior corporate governance provided larger dividends to their owners. Hypothesis 2b is also tested in model 2 of Table 4. The coefficient of the interaction term of CSR and CG index (CSRCG index) is positively significant at *p* < 0.01, with a coefficient value of 0.088. In economic terms, a CSRCG index increase of one standard deviation raises dividend payment by 82 percent. As a result, this finding confirms hypothesis 2b, indicating that the quality of corporate governance moderates the association between CSR and dividend distribution. The positive correlation between moderator and dividend pay-out reinforces the existing CSR–dividend pay-out link. These findings imply that CG quality has a considerable impact on mutual fund’s dividend pay-outs in Pakistan. Overall, the indications and significance of control variable coefficients in model 2 are similar to those in model 1.

Table 4 also shows the results of the frequency of dividend payments in models 3 and 4. The coefficient of CSR in model 3 is –2.337 significant at *p* < 0.01. In economic terms, a one-standard-deviation increase in CSR reduces the frequency of dividend payments by 15.39%. In model 4, CSR and CG_Index are positively significantly related to the frequency to pay dividend at *p* < 0.01. However, CSR × CG_Index is negatively significantly related to the frequency to pay dividend at *p* < 0.01. This means that funds with higher CSR activities have a lower frequency to pay dividend. These findings are in line with the cost of equity channel approach of CSR. It means that funds with higher CSR activities may prefer to hold cash and invest it to enhance the funds profitability, instead of paying dividend more frequently due to lower opportunity cost of hoarding cash. The higher corporate governance is strengthening the relationship between CSR and frequency to pay dividend. Overall findings of Table 4 suggest that funds with higher CSR activities pay more overall dividends; however, their frequency to pay dividend is lower as compared to lower CSR activities’ funds. Furthermore, corporate governance helps to reinforce the link between CSR and dividend pay-out by acting as a moderator.

### Additional analysis

According to the Mutual Fund Association of Pakistan (MUFAP), mutual fund industry has Rs. 982.3 billion of total assets under management till the end of May 2021. The mutual fund industry of Pakistan consists of both Islamic and conventional mutual funds. Both types of funds work in a similar manner; however, conventional funds are free to invest in stock and bonds, which could be redeemed at any time, have a higher risk, and return as compared to Islamic funds. On the contrary, Islamic funds are bound to make investments according to the principles of Islamic law, which promotes the concept of profit sharing instead of fixed returns in the form of interest. Therefore, Islamic funds are considered less risky, which is empirically confirmed by Naveed et al. (2020b) in the case of Pakistan. Subsequently, management of Islamic and conventional funds may adopt different pay-out policies to satisfy their investors, which may demand different required rates of returns due to different risk characteristics.

The Islamic religious concepts such as vicegerency, divine accountability, and the enjoying goods derivate the CSR under the Islamic paradigm. According to Farook (2007), Shariah financial institutions perform mandatory and recommended CSR activities. The mandatory responsibilities include screening the business activities as per Shariah laws, avoiding earnings from prohibited sources, equal and fair treatment with employees, responsible dealing with clients, and effective policy regarding zakat. In addition, Islamic institutions are free to carry on recommended CSR activities like conventional counterparts. Furthermore, Islamic institutions are held responsible for CSR under the broader notion of vicegerency that prohibits Islamic institutions management from overinvesting in CSR for the purpose of their own-reputation building (Jusoh et al., 2015). However, an agency view suggests funds’ managers may involve in aggressive CSR activities to achieve their personal goals such as self-reputation and empire building. Therefore, they may pay more dividends in response to masking the overinvestment in the CSR activities.

Due to various financial limitations and objectives, conventional and Islamic fund managers may act differently in the same situation (Naz et al., 2017). To please their shareholders, managers of both types of funds may make CSR investments in various patterns and devise distinct dividend pay-out plans. As a result, we anticipate that our baseline findings will be sensitive to the mutual fund type. We use a two-step system GMM approach to assess the association between CSR and dividend pay-out of Islamic and conventional funds to ensure the results are reliable. Models 1 and 2 of Table 5 show the outcomes of Islamic funds, whereas models 3 and 4 show the results of conventional funds. The significance of AR1 and AR2 and the insignificance of Hensen J. tests are consistent with GMM regressions’ goodness-of-fit assumptions.

**TABLE 5:** Islamic vs. conventional funds: dividend pay-out as dependent variable.

	(1)	(2)	(3)	(4)
	Islamic funds		Conventional funds	
Variables	Pay-out	Pay-out	Pay-out	Pay-out
L.Dividend_Frequency	0.190***	0.226***	0.080***	0.065***
	(14.188)	(8.036)	(8.446)	(5.672)
CSR	0.300***	4.878***	–0.154***	–6.689***
	(3.257)	(3.837)	(–3.292)	(–6.773)
CG_Index		0.087***		–0.038***
		(4.808)		(–3.027)
CSR × CG_Index		–0.179***		0.255***
		(–3.871)		(6.729)
Perf	0.197***	0.167***	0.068***	0.035***
	(24.033)	(12.380)	(12.277)	(5.002)
F_Size	–0.017***	–0.016**	–0.025***	–0.015***
	(–3.635)	(–2.447)	(–7.544)	(–4.119)
Exp_Ratio	–0.007	–0.005	–0.051***	–0.036***
	(–1.195)	(–0.795)	(–10.333)	(–10.223)
MGT_Fee	0.135***	0.097***	0.048**	0.038***
	(6.833)	(7.815)	(2.377)	(2.773)
M_Edu	0.043***	0.031***	0.037***	0.028***
	(4.187)	(4.387)	(2.788)	(3.949)
M_Exper	0.005***	0.005***	0.023***	0.016***
	(4.471)	(6.723)	(23.664)	(12.669)
F_Age	0.003*	0.009***	0.012***	0.007***
	(1.809)	(4.192)	(5.436)	(3.059)
Year effects	Yes	Yes	Yes	Yes
Constant	0.079	–2.172***	0.362***	1.250***
	(0.770)	(–3.933)	(6.720)	(3.570)
Observations	490	490	815	815
Number of Funds	85	85	100	100
AR1 (Pr > z)	–4.696 (0.00)	–4.803 (0.00)	–6.711 (0.00)	–6.403 (0.00)
AR2 (Pr > z)	–0.868 (0.385)	–0.601 (0.548)	–0.483 (0.629)	–1.057 (0.29)
Hansen J. (Prob > Chi)	60.27 (0.875)	52.06 (0.319)	81.44 (0.259)	79.09 (0.322)

z-statistics in parentheses: ***p < 0.01, **p < 0.05, *p < 0.1.

In model 1, the CSR coefficient is 0.300 significant at p0.01. In terms of economics, a one-standard-deviation increase in CSR boosts the dividend pay-out by 9.26%. The coefficient of CSRCG index in model 2 is—0.179 significant at *p* < 0.01. In terms of economics, a one-standard-deviation increase in the CSR × CG_Index reduces the dividend by 150 percent. Overall, a higher dividend payment by the Islamic funds in response to a high CSR is in the line of agency view; however, a significant reduction in the dividend pay-out ratio in Islamic funds with a higher governance score is in the line of expectation that the line of cost of equity capital channel of CSR that investors demand relatively lowers returns from less risky investments. In model 3, on the contrary, the coefficient is—0.154 significant at *p* < 0.01. In terms of economics, a rise in CSR by one standard deviation reduces the dividend by 5.13 percent. As a result of the substantial investment in CSR, traditional funds pay a lower dividend. This is in line with the empire-building perspective on agency conflict. In model 4, however, the coefficient of CSR × CG_Index is 0.255 significant at *p* < 0.01. These findings confirm the monitoring role of corporate governance in reducing agency conflict and predict a greater dividend payment in traditional funds with excellent corporate governance.

Table 6 reports the findings of Islamic and conventional funds using the frequency to pay the dividend as the dependent variable. The findings of GMM regressions suggest that both Islamic and conventional funds pay the dividend with a higher frequency in response to high CSR activities confirmed by the significant positive coefficients of CSR in model 1 and model 3, respectively. However, the coefficient of CSR (2.744) is higher in Islamic funds as compared to the value of coefficient (1.822) significant at *p* < 0.01. We also find the partial significance of CSR × CG_Index in the case of Islamic funds only in model 2. These findings suggest that funds with a higher CG index prefer the higher total dividend payment in a year as compared to paying the dividend with the maximum number of times in a year, particularly in conventional funds. Moreover, in terms of control variables, the significance and signs of coefficients are in the line of baseline regressions.

**TABLE 6:** Impact of corporate social responsibility (CSR) on frequency to pay dividend: Islamic vs. conventional funds.

	(1)	(2)	(3)	(4)
	Islamic funds		Conventional funds	
Variables	Dividend_Frequency	Dividend_Frequency	Dividend_Frequency	Dividend_Frequency
L.Dividend_Frequency	0.062***	0.073***	0.001	–0.008
	(7.959)	(4.880)	(0.107)	(–0.963)
CSR	2.744***	–3.172	1.822***	–3.044
	(6.847)	(–0.707)	(2.825)	(–0.604)
CG_Index		–0.007		0.078
		(–0.114)		(1.203)
CSR × CG_Index		0.312*		0.126
		(1.923)		(0.619)
Perf	0.498***	0.372***	0.416***	0.404***
	(14.459)	(9.230)	(6.455)	(6.249)
F_Size	0.174***	0.163***	–0.172***	–0.109***
	(8.139)	(6.696)	(–4.745)	(–3.281)
Exp_Ratio	0.138***	0.152***	–0.411***	–0.410***
	(10.085)	(6.379)	(–8.409)	(–13.092)
MGT_Fee	–0.181***	–0.141**	0.303***	0.268***
	(–5.261)	(–2.156)	(2.928)	(3.194)
M_Edu	0.121*	0.086	0.125	0.196**
	(1.905)	(1.076)	(1.245)	(2.416)
M_Exper	–0.004	–0.014**	0.219***	0.195***
	(–0.948)	(–2.181)	(16.303)	(15.529)
F_Age	–0.011**	–0.037***	0.061**	0.062***
	(–2.214)	(–4.766)	(2.437)	(3.882)
Year effects	Yes	Yes	Yes	Yes
Constant	–2.433***	–2.825*	1.490***	–0.782
	(–6.061)	(–1.645)	(2.623)	(–0.432)
Observations	490	490	815	815
Number of funds	85	85	100	100
AR1 (Pr > z)	–4.473 (0.00)	–4.748 (0.00)	–6.216 (0.00)	–6.186 (0.00)
AR2 (Pr > z)	–0.885 (0.376)	–0.940 (0.347)	–0.846 (0.398)	–0.700 (0.484)
Hansen J. (Prob > Chi)	54.42 (0.276)	41.68 (0.355)	55.51 (0.243)	72.72 (0.239)

z-statistics in parentheses: ***p < 0.01, **p < 0.05, *p < 0.1.

### Discussion

There has been considerable interest in corporate social responsibility (CSR) and socially responsible investing (SRI) during the past decade. Mutual funds have also seen tremendous growth and are touted as a viable alternative to equities, especially for individual and unsophisticated investors. Mutual funds appeal to these investors by providing a consistent dividend payment to their unit holders. Therefore, it is apt to study the impact of CSR engagement on dividend pay-out of mutual funds. Dividend policy may discipline the management and prevent the misuse of free cash flows in overinvestment that includes overinvestment in CSR. Corporate governance mechanisms are also designed to address the conflicts of interest and agency problems.

Considering the interest in CSR and investors’ perceptions, it can be safely assumed that CSR engagement would boost future cash flows and expected profitability. The findings are consistent with the market valuation approach, which shows that CSR participation is likely to boost the predicted cash flows and profitability (Sasongko et al., 2020), which can be distributed as dividend payments. CSR efforts can aid in gaining a competitive advantage, enhancing stakeholder relationships, improving management, and lowering transaction costs. Our results imply that mutual funds using SRI screening criteria are selecting firms for investment with a sustainable financial and profitability performance. Therefore, mutual funds are more likely to have a superior return performance and lower risk, which can translate into higher dividend payments. The results of the study also suggest that CG quality influences the dividend pay-out in line with the previous literature that has provided a positive link between CG quality and dividend pay-out at the firm level. Corporate governance quality of mutual funds also complements the CSR performance to positively impact the relationship between CSR performance and dividend pay-out of mutual funds. Asset management businesses should also be encouraged by policymakers and regulators to improve CG quality and engage in more CSR activities, which can contribute to improved fund performance and dividend distribution.

## Conclusion

Because mutual funds pay out dividends on a regular basis to their unit holders, they are viewed as a viable alternative to equities. Income payments are a concern for mutual fund investors who want to participate in funds that offer a bigger and more consistent dividend. The goal of this study was to see how CSR affects the mutual funds’ dividend pay-out. We also investigate the influence of CG quality on mutual funds, as well as the moderating role of CG in the CSR–dividend pay-out relationship. According to the forecast and past empirical studies, CSR engagement boosts future cash flows and predicted profitability, which can subsequently be transferred to shareholders in the form of dividend payments. CSR is favorably and strongly connected with the mutual fund’s dividend pay-out, according to the findings of the study. Furthermore, CG quality influences the dividend pay-out and positively moderates the association between mutual funds’ CSR and dividend pay-out. In addition, a thorough examination demonstrates that Islamic mutual funds with higher CSR and CG quality pay much higher dividends than traditional mutual funds. Conventional funds with excellent CG quality, on the contrary, offer much greater dividends when they invest more in CSR. The relationship is inverse in the case of Islamic funds.

Dividend payments are more important to mutual fund investors than fund performance. The funds develop a positive image and reputation in society by engaging in CSR activities and attracting investors to invest in a certain fund. Most mutual funds pay dividends to shareholders on a regular basis and delivering a greater income to shareholders attracts investors; as a result, fund managers prefer to pay a bigger dividend to entice investors to invest in a particular fund. Dividend pay-outs may help solve the agency problem between management and shareholders due to the limited quantity of cash flow available that may be exploited by self-interested managers. As a result, the dividend distribution policy may have a significant impact on management decisions and plays an important role in mutual fund monitoring. Policymakers and asset management organizations should take note of the findings. Because the study shows that CSR and CG have a beneficial impact on dividend payment, asset management businesses should strive to improve CG quality and participate in CSR activities to improve profitability, fund performance, and risk reduction. As a result, dividends can be increased, and asset management businesses can attract a larger pool of investors. Asset management businesses should also be encouraged by policymakers and regulators to improve CG quality and engage in more CSR activities, which can contribute to improved fund performance and dividend distribution.

The study has implications for regulators, policymakers, and investors of mutual funds in Pakistan. First, in the context of Pakistan where ownership is highly concentrated, firms indulge in CSR activities to gain legitimacy and maintain good relations with the stakeholders. In the context of mutual funds, asset managers can improve the dividend pay-out ratio by implementing a higher level of CSR activities. The funds can build a good image and reputation in society by investing in CSR activities and attracting investors for investment in a particular fund, as mutual funds’ investors are more concerned with the dividend pay-out. Second, fund managers who care about social responsibility should ensure effective corporate governance as it strengthens the relationship between CSR and dividend pay-out of mutual funds. The paper also offers insights to regulators and policymakers that corporate social responsibility and corporate governance quality matters for having a superior fund performance and higher dividend payments. As the corporate social responsibility can enhance the funds’ dividend pay-out, the regulatory bodies should pay attention to make the asset management firms to mandatory follow the social responsibility practices. Like the code of corporate governance in Pakistan, the regulatory bodies need to develop the social responsibility code, which would help the asset management firms enhance dividend payments.

The study’s weakness is that it is limited to a particular country and hence cannot be applied to other industrialized economies. Another limitation of the study is that we could not include the data for recent years because of the non-availability of the CSR data for more recent years and the laborious nature of the manual content analysis for CSR disclosure index. The future research could address this issue by including most recent data and examining the relationship between CSR performance and dividend pay-out of mutual funds in a cross-country context, which would allow generalizing the findings of the study.

## Data availability statement

The raw data supporting the conclusions of this article will be made available by the authors, without undue reservation.

## Author contributions

All authors listed have made a substantial, direct, and intellectual contribution to the work, and approved it for publication.

## Footnotes

1 Here, SECP denotes Securities and Exchange Commission of Pakistan.
